# Atypical leptospirosis: an overlooked cause of aseptic meningitis

**DOI:** 10.1186/s13104-016-1964-z

**Published:** 2016-03-10

**Authors:** Ning Wang, Yu-Hsuan Han, Jia-Ying Sung, Wen-Sen Lee, Tsong-Yih Ou

**Affiliations:** Department of Neurology, Wan Fang Medical Center, Taipei Medical University, No. 111, Section. 3, Shing Long Road, Taipei, 11696 Taiwan; Division of Infectious Diseases, Department of Internal Medicine, Wan Fang Medical Center, Taipei Medical University, No. 111, Section. 3, Shing Long Road, Taipei, 11696 Taiwan

**Keywords:** Leptospirosis, *Leptospira*, Aseptic meningitis, Neuroleptospirosis, Zoonosis

## Abstract

**Background:**

Leptospirosis, probably the most common zoonosis in the world, is caused by pathogenic *Leptospira* species. Clinical presentations range from nonspecific fevers to fulminant diseases such as Weil’s syndrome. Neurological forms of leptospirosis (neuroleptospirosis) are usually underestimated, and many cases of leptospirosis are overlooked because of the lack of specificity of signs and symptoms. Diagnosis confirmation is difficult because of the challenges associated with isolating the organism and positive serologic testing. A comprehensive understanding of the clinical presentation of leptospirosis and risk factors for exposure to leptospirae are required for early diagnosis, in order to initiate appropriate treatment immediately.

**Case presentation:**

Here we present one male patient with anicteric leptospirosis that manifested as neuroleptospirosis with aseptic meningitis, although he did not have impaired kidney function or thrombocytopenia. He recovered well after an early investigation and treatment for leptospirosis based on suspected relevant risk factors and clinical manifestations.

**Conclusion:**

To facilitate optimal use of antibiotic treatments and prevent lethal complications of leptospirosis, we report this case of leptospirosis, which highlights the importance of knowing the occupational history and environmental exposures of patients living in leptospirosis-endemic areas and presenting meningeal signs.

## Background

Leptospirosis is an acute febrile zoonotic disease caused by infection with pathogenic spirochetes of the genus *Leptospira*. It is transmitted to humans principally via environmental water contaminated with the urine of wild and domestic mammals that are chronically colonized by *Leptospira* [1]. The leptospires dwell in the renal tubules of their animal hosts, including almost all known species of rodents, marsupials and other mammals, which are common carriers and excretors of leptospires [2]. Risk of infection is associated with occupational and recreational activities, as well as household environments where there is close contact with animals. The clinical presentations are diverse, ranging from undifferentiated fever to fatal disease. Anicteric leptospirosis is the most common manifestation, occurring in 80 % of cases [3]. Its diagnosis relies upon a strong suspicion of this disease based on various nonspecific clinical presentations. This worldwide disease has been found to occur predominantly in wet tropical and subtropical regions [4, 5], including Taiwan. Here, we describe a male patient with leptospirosis, who presented with aseptic meningitis but without jaundice, impaired kidney function or thrombocytopenia. The patient was prudently investigated and treated based on his relevant exposure history and clinical manifestations.

## Case report

The 37-year-old male patient was a cook working in a restaurant close to a waste recycling station and a pond for recreational shrimp fishing. The patient was generally healthy and had suffered no major illnesses until 1 week before hospitalization. At that time, in late November 2012 (autumn), he presented with a low grade fever, nausea, and vomiting, which subsided rapidly within 2 days after taking prescription medicines containing no antibiotics or nonsteroidal anti-inflammatory drugs. He returned to work shortly thereafter, but soon the abrupt onset of persistent diffuse headaches and a sensation of fullness resulted in disturbed sleep. He experienced unrelenting vomiting, and in addition he recalled that he had persistent neck stiffness, even when he lay down. He was then admitted to the emergency unit, at which time his blood pressure was 129/77 mmHg, body temperature was 37 °C, pulse rate was 93 beats/min, and respiratory rate was 20 times/min. The physical examination showed that he had an alert, distressed facial appearance, with anicteric sclerae and a supple neck without lymphadenopathy. He did not have any neurologic deficits or skin abnormalities. He did not show any abnormalities in other system examinations. The patient denied any history of recent travel, but mentioned that his working environment was infested with rats, which ran through the kitchen in the daytime.

The initial laboratory examination of peripheral blood showed that his serum haemoglobin level was 15.7 g/dl, platelet count was 338,000 cells/μl, white blood cell (WBC) count was 18,670 cells/μl with 81.2 % segmented neutrophils and 14.1 % lymphocytes, his C-reactive protein was 20 mg/dl, creatinine was 1.03 mg/dl, aspartate aminotransferase (AST) was 34 IU/l, and glucose was 117 mg/dl. The initial lumbar puncture revealed an opening pressure of 24 cmH_2_O. The cerebrospinal fluid (CSF) tested negative for the cryptococcal antigen test and India Ink test, and had a WBC of 254 cells/μl comprising 91 % mononuclear cells and 9 % segmented neutrophils, a red blood cell count (RBC) of 36 cells/μl, total protein of 91 mg/dl, and glucose of 64 mg/dl. Based on these initial laboratory results, the patient received 2 g of ceftriaxone q12h and 500 mg of acyclovir q8h for meningitis, including aseptic meningitis.

Despite the identification and treatment of aseptic meningitis, his clinical condition did not improve. He was further investigated for other pathogens that might cause aseptic meningitis. Upon re-evaluation of his clinical presentations, with a focus on his occupation and hobby of shrimp fishing, the causal pathogen was suspected to be microbes that could cause CSF pleocytosis, apparently as a result of aseptic meningitis. After undergoing additional tests for atypical pathogens in the serum, such as the latex agglutination test for cryptococcal antigen, particle agglutination assay for IgG and IgM antibodies to *Mycoplasma pneumonia*, and an electrochemiluminescence immunoassay for IgG and IgM antibodies to *Toxoplasma gondii*, he received additional minocycline, ampicillin and steroids for his unremitting increased intracranial pressure-induced headache and vomiting.

His clinical symptoms improved markedly after day two. The follow-up lumbar puncture on hospital day four revealed an opening pressure of 23.6 cmH_2_O, a WBC of 266 cells/μl comprising 99 % mononuclear cells, a RBC of 141 cells/μl, total protein of 33 mg/dl, and glucose level of 78 mg/dl, compared with a serum glucose level of 141 mg/dl. Other follow-up laboratory test results were unremarkable, except for a WBC of 14,840 cells/μl with 83 % segmented neutrophils and 12 % lymphocytes. Ceftriaxone was discontinued, despite no evidence of pneumococcus or enteric rods. The aforementioned serological tests for atypical pathogens revealed negative results for *Cryptococcus*, mycoplasma and toxoplasma. The particle agglutination assay for HIV antibody in the serum sample and the polymerase chain reaction (PCR) method for detecting tuberculosis and the herpes simplex virus also showed negative findings. The Taiwan Centers for Disease Control laboratory performed an indirect immunofluorescence assay for Q fever, scrub typhus and epidemic typhus with paired serum samples, and PCR testing of whole blood samples. These samples were collected on hospital days 2 and 16.

Acyclovir was discontinued on hospital day 10, since there was no evidence of a herpes simplex viral infection. On hospital day 14, the patient’s third lumbar puncture showed an opening pressure of 18 cmH_2_O, a WBC of 49 cells/μl with 99 % mononuclear cells, a RBC of 1 cell/μl, total protein of 32 mg/dl, and a glucose level of 81 mg/dl, compared with a serum glucose level of 153 mg/dl.

The patient was discharged on hospital day 16 after being treated with minocycline and ampicillin for 14 days. On the day after discharge, we received confirmation from the Taiwan Centers for Disease Control of the diagnosis of leptospirosis via a microscopic agglutination test (MAT), with convalescent titers of *L. interrogans* serovar bataviae and *L. santarosai* serovar Shermanii at a 1:100 dilution and a 1:800 dilution, respectively, although the titers were both negative in the initial acute phase. The patient was therefore diagnosed as having been infected by *L. santarosai* serovar Shermanii with meningitis.

## Discussion

We have presented the case of a male patient with a mild form of leptospirosis. The interesting aspect of this patient was an isolated central nervous system involvement, in the form of aseptic meningitis. He did not show any of the other more common symptoms of leptospirosis, such as myalgia, conjunctival suffusion, jaundice, thrombocytopenia, or abnormalities in the hepatic or renal functions. The rat infestation at his workplace was the only clue, in addition to a strong suspicion of this zoonotic disease, that allowed us to distinguish it from common viral meningitis.

Leptospirosis in humans is acquired through contact with the urine or tissues of an infected animal, or through contaminated water and soil [5]. Rodents are recognized as the most important sources of the transmission of leptospirosis [5]. Risk factors for exposure to leptospirae include occupational exposure (farmers and ranchers, abattoir workers, trappers, veterinary surgeons, loggers, sewer workers, rice-field workers and military personnel), recreational activities (freshwater swimming, canoeing and kayaking, trail biking and hunting) and household environmental factors (domestic dogs and livestock, rainwater catchment systems and rodent infestations) [4].

During the bacteraemic phase, leptospires can be cultured from the blood, CSF, and other tissues, but not from urine [6]. Leptospiruria can persist in humans for 1–3 weeks, and in some animals leptospires can be shed chronically [7]. During the immune phase, lymphocytic pleocytosis occurs, with total cell counts usually below 500 cells/μl, and diagnosis can be made through immunological tests [7]. In this later phase, the CSF is characterized by protein levels of 50–100 mg/dl and generally normal glucose concentrations [7].

As stated previously, aseptic meningitis with or without symptoms is characteristic of the immune phase of the illness, occurring in up to 80 % of patients [7]. In endemic areas, a significant proportion of all aseptic meningitis cases may be caused by leptospiral infection [7]. Severe neurologic complications, such as coma, meningoencephalitis, hemiplegia, transverse myelitis or Guillain-Barré syndrome, occur only rarely [5, 7]. Leptospirosis is responsible for 5–13 % of all cases of aseptic manifestations, including hemiplegia, intracranial bleeding, cerebellitis, movement disorders, myelitis, and acute flaccid paralysis such as Guillain-Barré syndrome, mononeuritis, facial palsy, and neuralgias [8]. Meningeal signs occur in 80 % of cases [7], but leptospirosis rarely manifests primarily as a neurological disease [9]. Wang et al. [10] reported a case of a 44-year-old male leptospirosis patient who presented with neuroleptospirosis with transient paraparesis, thrombocytopenia, mild elevated aminotransferase, mild bilirubinemia (total bilirubin 2.0 mg/dl) and mild impaired kidney function (serum creatinine 2 mg/dl), but without meningitis. Our patient, who had risk factors associated with exposure to rats and his occupation as a cook, presented with aseptic meningitis with normal liver and kidney function. Our CSF culture showed no growth, which could be explained by a recall bias for the exact time of symptom onset. However, since we strongly suspected leptospirosis, we prudently investigated that possibility and treated him, and he recovered well without sequelae.

Taiwan lies in the path of many tropical storms and typhoons, which bring extremely heavy rainfall, usually in July–September. The mean annual incidence of leptospirosis is 0.21 cases/100,000 people [11]. The major serotype thus far identified has been *L. santarosai* serovar Shermanii [11, 12]. Acute disseminated encephalomyelitis has rarely been reported following leptospirosis [13]. In cases of aseptic meningitis occurring in areas with endemic leptospirosis, using a modified Faine’s criteria score in a preliminary evaluation of the patient [14] may be helpful for further treatment decisions and in investigating the possibility of leptospirosis. Magnetic resonance imaging (MRI) is a good method for identifying acute disseminated encephalomyelitis [13]. Our case was only evaluated via computed tomography (CT), so we could not confirm or exclude encephalitis.

The present case was diagnosed as neuroleptospirosis based on CSF pleocytosis and positive MAT results for acute and convalescent serum. We were fortunate to obtain a positive result for the paired serum using the MAT serology method. Several recent publications reveal that the molecular methods for detecting nucleic acids have improved in diagnostic sensitivity. A quantitative PCR targeting the pathogenic *Leptospira*-specific 16S ribosomal RNA gene was found to be a promising tool for the rapid diagnosis of acute leptospirosis, with a wide temporal window for the detection of positive results [15]. The Lepto-MD assay, which targets the *Leptospira* 16S *rrs* gene and can be performed as a real-time RT-PCR, has demonstrated an increased sensitivity in *Leptospira* detection. This molecular method shows virtually no overlap with the single-specimen MAT serology method, and a combined testing strategy for acute leptospirosis could maximize case detection [16].

Two cases have been diagnosed with neuroleptospirosis based on *Leptospir*a DNA that was detected in the CSF. In one of these two patients, the DNA was detected only in the CSF, in the absence of leptospiremia [17]. Unbiased next-generation sequencing of the CSF and serum was used to diagnose primary neuroleptospirosis of *Leptospira santarosai* in a 14-year-old boy with severe combined immunodeficiency (SCID), who had received a bone marrow transplant. He was not diagnosed over a period of 4 months, during which he experienced progressively worse symptoms, including hydrocephalus and status epilepticus, until the next-generation sequencing method detected *Leptospira* DNA in his CSF but not in his serum. This approach thus facilitated the use of targeted and efficacious antimicrobial therapy [18].

## Conclusion

In order to facilitate optimal use of antibiotic treatment and prevent the lethal complications of leptospirosis, we have reported this case of a patient with leptospirosis, because it highlights the importance of knowing the occupational history and environmental exposures of patients living in leptospirosis-endemic areas, who present with meningeal signs.

### Consent

Written informed consent was obtained from the patient for publication of this case report and any accompanying images.

## Abbreviations

AST: aspartate aminotransferase
CSF: cerebrospinal fluid
CT: computed tomography
MAT: microscopic agglutination test
MRI: magnetic resonance imaging
PCR: polymerase chain reaction
RBC: red blood cell
WBC: white blood cell
